# Latent Profiles of Deceased Organ Donation Registrants and Nonregistrants in the United States

**DOI:** 10.1155/joot/4446435

**Published:** 2025-07-18

**Authors:** Ari P. Kirshenbaum, Brendan Parent, Landry Goodgame Huffman, Virginia Kelsey, Michael J. Sofis

**Affiliations:** ^1^Department of Innovation, Advocates for Human Potential, Sudbury, Massachusetts, USA; ^2^Department of Psychology, Saint Michael's College, Colchester, Vermont, USA; ^3^Department of Population Health, Division of Medical Ethics, NYU Grossman School of Medicine, New York, New York, USA

**Keywords:** deceased organ donation, latent profile analysis, registration, young adults

## Abstract

Deceased organ donation is the greatest source of transplantable whole organs, but registration rates are a limiting factor because they remain low among certain populations. A stratified, nonprobability survey was used to identify population characteristics associated with nonregistration in the United States (*N* = 11,083). Latent profile analysis (LPA) was used to identify multivariate patterns of demographic, socioeconomic, and health-related factors associated with registration. LPA yielded three distinct profiles, which all reported similar average percentages of driver's license possession, medical insurance coverage, and income, indicating that profiles were not distinguished by these variables. Meaningful differences across the profiles included access to healthcare services, satisfaction with those services, general health and well-being, and age; those who are both healthy and young (mean age = 25.9 years) reported the lowest percentage of organ donation registration (35.3%). For this group, 71.48% listed either low priority or distrust in the donation process as the top reasons for nonregistration. Importantly, age as a standalone variable was not uniformly associated with donation and was conditionally dependent upon health status; poorer health in young adults was associated with greater registration. These findings reveal previously unidentified opportunities for tailoring donor registration campaigns to populations with a high potential for registration behavior change.

## 1. Introduction

Recent estimates (March 2024) demonstrate that 17 people die each day in the United States waiting for an organ transplant, and there are currently 103,223 on the national transplant waitlist [1]. Increasing the rate of registration for deceased organ donation is among the greatest opportunities to meet shortages in transplantable solid organs. Despite this, state policies to promote deceased organ donation have fallen short of significantly increasing registration [2], and in fact, there appears to be a decline in the overall percentage of United States' citizens registering [1]. Sociodemographic patterns associated with registration remain unclear [3], but the age of the registrant may play an important factor [4, 5]. Lower rates of registration among transition-aged youth and young adults may be related to a lack of education [6] or potentially limited reliance on the healthcare system. Primary care has been identified as an important touchpoint to promote the awareness of the medical need for transplantable organs [7].

Although these trends provide valuable insights, they are limited by their focus on mean-level characteristics, such as correlating average rates of donor registration with age. These variable-centered analyses often create an oversimplified picture of organ donation and fall short of accommodating the wide range of potential factors and high intraindividual variability that likely contribute to the decision to register. These factors include differences in demographics, socioeconomics, mental and behavioral health issues, and experiences with healthcare. Other statistical methods such as latent profile analysis (LPA) are better suited to characterizing this high multivariate heterogeneity between and within individuals. LPA is an unsupervised machine-learning approach that creates profiles (a.k.a. clusters or subgroups) based on the distinct patterns among multiple variables. A recent study by Gong et al. [8] highlighted the utility of LPA in identifying the multivariate associations between media use and willingness to donate and accept organs. Specifically, the authors found that those with “occluded” media use, or media exposure that was largely limited to TV and radio, were far less likely to donate and accept organs than those who used smartphones more frequently than any other media. The use of LPA in that study provided a robust opportunity to explore the complex effects of both media use frequency and type on organ donation.

This survey study utilized LPA to gain a current understanding of sociodemographic patterns associated with deceased organ donor registration and to elucidate the degree to which involvement and satisfaction with the healthcare system moderates registration. The reasons for nonregistration were also assessed to provide insight into which attitudes are outstanding obstacles toward registration.

## 2. Methods

### 2.1. Survey Methods

Data for this study were collected for all 50 states between October and November of 2023 as a part of an ongoing national survey called the National Behavioral Health Survey (NBHS). Research panels from the Cint Digital-Insights Gathering Platform were used to recruit participants 16 years and older. This was a nonprobability, purposive sampling technique in which we used concurrent demographic quotas (e.g., % reporting per state, % gender balance, etc.) to guide sampling. Information statements, including informed consent requirements, were presented to participants immediately upon arrival at the survey landing page and a computer bot check was used to screen participants. Participants were paid approximately $1–4 for survey completion, although the exact form and amount of payment are not available to precisely calculate from the research panels. The average completion time for this survey was approximately 19 min. The Institutional Review Board at Advocates of Human Potential (Federal Wide Assurance #6316) approved all procedures and activities of this study (IRB#00000984).

### 2.2. Survey Measures

Included in the survey were standard demographic questions on income, age, and race/ethnicity, based on the Department of Health and Human Services designations. Participants were asked to provide ZIP codes, which permitted a state-level analysis of how financial incentive programs for living-organ donation might influence registration for posthumous registration. This was used in the analysis to determine whether an awareness of state-level insurance protections for living donors could positively impact registration for deceased donations.

Health and well-being survey items included the two most reported mental health illnesses to assess population well-being. The Generalized Anxiety Disorder 2-item scale (GAD-2) [9] and the Patient Health Questionnaire (PHQ-2) [10] which assesses symptoms of major depression were used. To assess current physical health, we used a measure of chronic pain, which was measured on a 10-point visual analog with the survey item “During the past 7 days, what number best describes how pain interfered with your general activity, on a scale from 0 = does not interfere to 10 = completely interferes?” This was adapted from the Pain, Enjoyment of Life General Activity Scale (PEG-3) [11]. Chronic pain tends to be a good indicator of overall health and well-being [12].

Since interactions with the healthcare system were particularly relevant to this study, we assessed access to, and satisfaction with, healthcare. Healthcare satisfaction was measured using a five-item Likert scale by “Think about the most recent time you received care from a physician or other medical professional within the past 12 months. Please indicate how satisfied you were with the quality of the care.” Ease of healthcare access was measured by “In the past 12 months, how easy or difficult was it for you to access healthcare services when you needed them?” This was a five-item Likert scale. Obstacles to healthcare access were assessed by “In the past 12 months, think about any and all of the health conditions you have been dealing with or have concerns about. Did you experience the following barriers when you tried to access treatment for a health condition?” This was a yes/no response to nine items which included “too costly,” “lack of transportation,” and “fear of stigma or being judged by the provider.” Medical contact was measured by the sum of three questions, each on a five-point scale from “never” to “always,” these being the frequency of going to a doctor for the treatment of illness, following a physician's treatment plan, and using a prescription as prescribed. A question about current medical insurance coverage (yes/no) was also included.

Organ donation questions occurred at the end of the survey. Those indicating “no” to “Are you a registered organ donor?” were prompted to answer an additional multiple-choice question regarding the reasons for not donating, and they could select more than one. These included “I want my body to be whole after I die” and “my religion does not permit it,” among others. Those who answered “unsure” to the question on organ donation registration were excluded from the survey data. A single-item question on the frequency of charitable giving was used to determine whether organ donation registration was related to a general tendency to give to others; this item appeared earlier in the survey and was therefore separated from the organ donation questions. This question was included to determine whether general prosocial tendencies were systematically related to donor registration.

### 2.3. Data Analysis

Registration percentages, per state, were examined using a heat map to provide a graphical description of geographic differences in registration. To determine whether our survey results were congruent with extant data on actual deceased donations, per state, a correlation was performed with our survey results and data obtained from the Procurement and Transplant Network (https://optn.transplant.hrsa.gov).

LPA was used to identify multivariate patterns of demographic, socioeconomic, and health-related factors associated with organ donor registration. All continuous and categorial variables used as profile indicators are listed in Supporting Information. To determine the optimal number of profiles, we began with a null 1-profile model and increased the number of profiles by one until the stopping criteria were reached. BIC and SABIC in past simulation studies have indicated that these predict the optimal number of profiles with significantly greater accuracy compared to other fit indices including AIC, entropy, VLMRT, and BLRT. As such, BIC and SABIC were used to determine whether the *k*-profile solution was better than the *k* − 1 profile solution, indicated by decreases in both values. We also included as a stopping criterion a profile size of less than 5% of the sample to encourage model parsimony and maximize qualitative differences (in both item response probabilities and probabilities of latent profile membership) between profiles. Finally, we did not estimate the *k* + 1 model if the log-likelihood was not replicated or the model did not converge, both of which are indicative of poor fit and model nonidentification. After establishing the optimal profile solution, we explored differences in demographic covariates and frequency of registration using the method developed by Lanza et al. [13], in which, the distal outcome is used as a latent profile predictor in a multinomial logistic regression alongside the latent profile model. Simulation studies have established this method as one of the best approaches for estimating the associations between latent profile membership and distal outcomes [14]. Reasons for not donating were assessed using *X*^2^ tests and Cramer's *V* tests to compare between-profile percentages reporting “yes” for each question.

Logistic regression was used to examine how demographic variables contribute to donation registration. Information obtained from such a regression may help to identify certain populations which may be critical to address with messaging. Also, driver's license ownership and insurance coverage were included since the opportunity to register as a donor can occur during the application process for both. The frequency of charitable giving was also included in the model to determine whether organ donation registration is related to a general tendency, or personality trait, to be generous to others. All statistics were performed in SPSS (V.29.01) and Mplus (V.8.10), and GraphPad Prism (V.10) was used to create figures.

## 3. Results

### 3.1. Overall Sample

**Figure 1:**
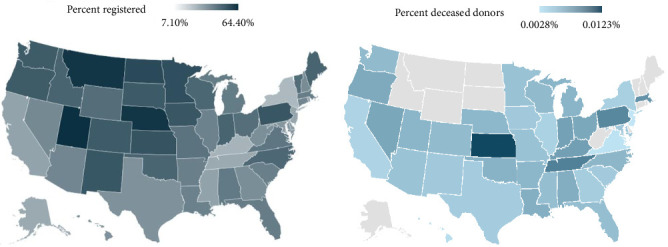
(a) Registration percentages per US state in the current survey compared to (b) deceased donors by state from the Organ Procurement and Transplant Network. Note: Some state data were not available via the Human Resources and Services Administration. These are shaded gray (b).

A total of 13,561 were recruited for the survey, but 1117 reported being “unsure” about whether they were registered donors and were excluded from the analyses, and 1361 failed to complete all survey measures. The resulting *N* = 11,083 and demographics are listed in Table 1 relative to the United States' Census (2020).  Figure 1 shows a heat map of donation registration percentages, per state. Average reported deceased donations from 2020 to 2024 (Organ Procurement and Transplant Network, https://optn.transplant.hrsa.gov) correlated with state percentages of registered organ donors in the survey (*ρ* = 0.35, CI: 0.02–0.61, *p* < 0.05).

### 3.2. Latent Profiles

**Figure 2:**
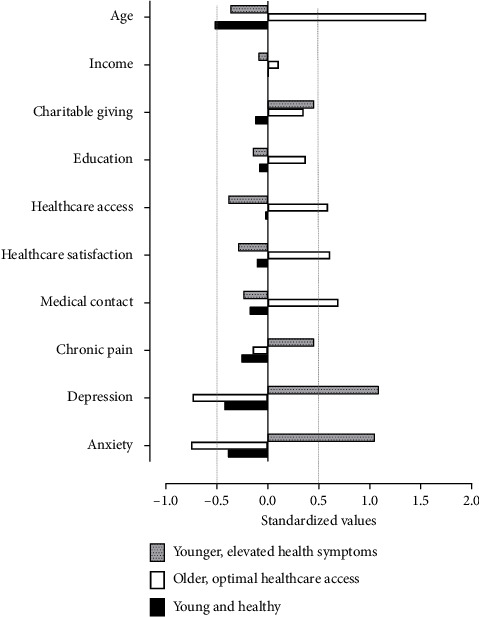
Continuous variables from the latent profile analysis for each group, relative to the grand means for all groups.

The LPA indicated an optimal fit of three distinct population profiles, and each was associated with a different rate of donor registration; LPA fit indices are listed in Supporting Information. The profiles have been labeled as follows, and in the tables and figures, according to their quantitative differences pertaining to age and healthcare utilization. Demographic characteristics associated with each profile are listed in Table 2. Continuous variables that separated the profiles are depicted in Figure 2, which shows that age varied significantly across the profiles, but income did not.

  Profile 1, or the younger and healthy group is associated with the lowest rate of registration (35.3%). They are younger (25.9 years) and more physically and mentally healthy when compared to the other groups (see Figure 2). Their income, education level, access to, and satisfaction with healthcare services are on par with the other two profiles (Table 2). This profile is also characterized by the highest proportion of males. Of those in this lowest-registration profile, 44.4% reported that their reasons for nonregistration included “Have not gotten around to registering.” Table 3 displays *X*^2^ and Cramer's *V* analyses results along with the percentages from other groups regarding the other reasons for not registering.
  Profile 2, or older, optimal healthcare access group, is associated with the highest rate of organ donor registration (47.5%) and is much older than the other two profiles (63.7 years) (see Figure 2). They are distinguished by above-average (> 0.5 standard deviations) utilization of healthcare services, as might be expected for this age group, and they are relatively satisfied with the quality of healthcare services they receive. They reported lower levels of both anxiety and depression symptoms, so they appear to have better mental well-being than those in the other two profiles. They are slightly higher in both income and total years of education (< 0.5 standard deviation from average) and are mostly white (> 80%) and are more likely to have medical insurance and a driver's license (Table 2). For those within this group who have elected not to register, their top reason (28.9%) was “I do not think anyone would want my organs” (see Table 3).

The younger, elevated health symptoms group, *Profile 3*, is also characterized by a relatively high rate of donor registration (43.7%). They are less healthy than those in Profile 1, who are similar in age. They exhibit elevated symptoms of depression and anxiety and report more chronic pain than those in either of the other profiles (Figure 2). They are also more dissatisfied with the healthcare they receive and report a lack of healthcare access. Those in this profile are not significantly lower in terms of education nor income relative to the other two profiles. The members of this profile reported giving to charities more frequently than either of the other groups.

The results of the logistic regression are presented in Table 4. The variables which most strongly predicted donation registration were self-identifying as white, having a driver's license, having reached a higher level of education, and being medically insured. Male identification was negatively predictive. There was no predictive relationship between identifying as Hispanic in our sample, nor was the frequency of charitable giving. Income was marginally related to registration. Age was also unrelated to registration; although this may seem to contradict the LPA, the influence of age on registration appears to be conditionally dependent on health status, as the differences between Profiles 1 and 3 suggest.

## 4. Discussion

The profile associated with the lowest percentage of deceased donor registration included the youngest survey respondents who were healthier compared to their counterparts in the other profile groups. The top reason for nonregistration in this “young and healthy” profile was low prioritization, procrastination, or apathy (i.e., “I have not gotten around to registering,” Table 3). Compared to their older counterparts, this group of younger respondents has experienced fewer opportunities to register, such as those who accompany driver's license renewals. Overall, the youngest, healthiest profile is more resistant to register for a variety of reasons listed in the survey; so, there are consistent obstacles toward registration for those among this group which helps to contextualize their low registration.

The second most cited reason for nonregistration in Profile 1 is distrust in organ donation. Worth noting is that both distrust and low prioritization/procrastination are obstacles which are distinct from the others listed in Table 3 in that they present opportunities for change. The other reasons for rejecting donor registration appear less malleable to the influence of public health messaging campaigns. Given that this profile of young and healthy individuals was associated with the lowest rate of donor registration, direct educational messaging about organ donorship could yield a substantial change in the proportion of the United States' population who may register. This group is not considered a protected or vulnerable population, and as such, there are fewer ethical concerns about coercion; guidelines for using social media to promote organ donation are available [15], and social media may be the best resource for targeted messaging campaigns in this age group.

Increased utilization of healthcare services that accompanies aging may be one-factor governing age-dependent differences in organ donation registration [16, 17]. The LPA demonstrates that favorable interactions with the healthcare system are sufficiently, but not necessarily, associated with greater percentages of donor registration (Figure 2). We say this because healthcare access and satisfaction failed to play a uniform role in those profiles who were most likely to donate: the older, optimal healthcare and younger, and elevated health symptoms profiles, which showed notable differences in healthcare satisfaction, failed to differ regarding the occurrence of registration. At least for the older group, who may interact with healthcare more regularly or incidentally, medical contact appears correlated with donor registration. For those in the younger group with elevated symptoms of chronic pain and emotional disturbances, healthcare satisfaction seems unrelated to their willingness to donate organs; this group's interest in donation could be more closely aligned with their reported charity which distinguishes them from the other two profiles. Among younger demographics, higher rates of empathy are a significant predictor of young adults' willingness to donate organs [18, 19], so our finding involving charitable donations within this profile is consistent with previous surveys.

Overall, the percentages of registration reported in our sample are consistent with current estimates from other national samples within the past year [20] and our sample correlates with deceased donations (by state) reported by the Health Services and Resources Administration (Figure 1). Both groups of younger survey respondents (i.e., Profiles 1 and 3) demonstrate a distrust of the organ-recovery system, and our data on younger adults are aligned with previous findings [17, 21, 22]. Among young adults, there is a discrepancy between expressed willingness to donate and the amount of those actually registered as donors [23, 24]. Within this age group of adults under 30 years, the intention-action gap and/or psychological inertia has been cited as a central obstacle to registration [17, 25]. In addition, a lack of discussion with family has also been identified as a challenge for this age demographic [23], and these important conversations tend to be delayed or dismissed because of tendencies to avoid interpersonal conflict [26]. Furthermore, our results echo previous findings in that the highest level of education achieved is positively associated with registration, and that misconceptions regarding donation remain significant barriers to registration [23]. Distrust of organ donation, especially among the younger age groups, may be related to misinformation presented via various media sources which is designed to attract attention from adolescents. Younger populations who are on the cusp of registration eligibility have reported that their media exposure is their primary source of information about organ donation [27], and television dramas have been explicitly cited by these groups as exaggerating negative donation outcomes [24].

The growth of organ donor need in the last two decades in the United States parallels population growth among one particular demographic, these being Hispanic Americans [28]; yet reports have identified particularly low donor-registration rates among those in this ethnic demographic [16, 21]. The profile group which involved the highest percent registration in the current study also happened to coincide with the lowest percent of Hispanics; however, self-identifying as Hispanic in our sample failed to serve as an independent and significant predictive variable (Table 4). In terms of racial demographics in our sample, self-identifying as white was a robust positive predictor of donation. Previous reports on the willingness to donate among racial-minority groups have shown that willingness to donate organs is heavily influenced by the awareness of need, religion, and pressure from family [28]. Indeed, both profile groups of younger survey respondents which included similar proportions of ethnic and racial minorities indicated similar reasons for not registering, which included religion, pressure from others, and a distrust in organ donation.

In our sample, the highest level of education achieved was positively related in an orderly way with registration (Table 4). Some could argue that formal education appears to bolster the awareness of organ needs. However, given that the two younger profiles which corresponded with significantly different rates of registration were similarly educated (Table 2 and Figure 2), some variables other than formal education must be operating to enhance donation in younger adults. Furthermore, these two groups had similar low access and satisfaction with healthcare. What may be surmised from the greater percentage of registration in the “younger, elevated health symptoms” profile is that their higher degree of chronic pain coupled with more symptoms of mental illness may make them more attentive to the eventual end of life. Therefore, mortality saliency [29] may play a favorable role in the willingness to donate organs among younger populations. To the best of our knowledge, whether mortality saliency can be leveraged to improve rates of donation among the young and healthy remains to be tested in messaging campaigns.

The sample for this study falls within the range of most United States census demographics, so we have reason to believe that it represents a reasonable cross-section of the national population despite our sample having a lower median income. There are other limitations with our survey design which are worth noting, such that our questions pertaining to healthcare access, satisfaction, and medical contact are not validated measures. Furthermore, the main outcome variable (i.e., deceased donation registration) could not be objectively verified.

This survey represents the most recent United States representative sample describing donation registration and related barriers. A little under half of all United States citizens remain unregistered [1]. Increasing registration remains one of the most promising methods for reducing the burden of organ failure. While several organizations and programs exist to increase donor registration, the total number of registered donors has not significantly increased. It is possible that designing education and registration opportunities tailored to populations that demonstrate the greatest opportunity both in numbers and behavior modifiability could make a significant difference in registration outcomes, and thus future solid organ availability. Registration campaigns designed with attention to this survey's findings and tailored to regional needs should be implemented under a research framework to measure impact and be refined, accordingly.

## Figures and Tables

**Table 1:** Demographics of the survey sample relative to the most recent United States' census.

Population characteristics	United States' census	Overall sample
*n*		11,083
Mean age	39	34.89
Median income	$69k	$55k
Male	50%	45.65%
Transgender male	—	0.29%
Female	50%	49.87%
Transgender female	—	1.29%
Nonbinary	—	1.79%
American Indian or Alaskan native	1%	4.44%
Asian	6%	6.55%
Black	14%	20.26%
White	76%	66.38%
Native Hawaiian or Pacific Islander	< 1%	1.22%
Hispanic	19%	17.75%

**Table 2:** Profile demographics by percentages and means.

	1. Younger and healthy	2. Older, optimal healthcare access	3. Younger, elevated health symptoms
*n*	4953	2516	3614
Registered donor	35.3%	47.5%	43.7%

*Demographics*			
Mean age	25.9 years	63.6 years	28.6 years
Male	51.0%	42.7%	37.2%
Transgender male	0.8%	0%	2.7%
Female	45.0%	57.2%	55.7%
Transgender female	0.2%	0%	0.4%
Nonbinary	1.3%	0%	3.4%
American Indian or Alaskan native	2.5%	1.0%	2.7%
Asian	6.9%	2.3%	4.0%
Black	21.1%	8.2%	19.5%
White	56.8%	84.7%	66.3%
Native Hawaiian or Pacific islander	0.8%	0%	0.6%
Hispanic	21.5%	4.1%	20.7%
Mean income^∗^	$62,706	$67,817	$57,383
Education^∗^	5.4	6.4	5.2

*Other categorical indicators in latent profile analysis*			
State incentive for living donation	42.8%	37.7%	40.6%
Possess a driver's license	80.9%	94.2%	77.9%
Medically insured	80.9%	95.3%	77.2%

*Note:* Income was recorded as follows: Less than $10K = $5K; $10K–$19,999 = $15K; $20K–$29,999 = $25K; $30K–$39,999 = $35K; $40K–$49,999 = $45K; $50K–$59,999 = $55K; $60K–$69,999 = $65K; $70K–$79,999 = $75K; $80K–$89,999 = $85K; $90K–$99,999 = $95K; $100K–$149,999 = $125K; $150K–$199,999 = $175K; $200K or higher = $250K. Education was recorded as follows: Never attended school = 0; kindergarten–8th grade = 1; some high school, no diploma = 2; high school graduate = 3; high school equivalency = 4; trade school following high school = 5; some college, no degree = 6; associate's degree = 7; bachelor's degree = 8; master's degree = 9; doctoral degree = 10.

^∗^Income and education response options were consolidated to increase the ease of survey responding.

**Table 3:** Reasons for not registering as a donor, by profile.

	1. Younger and healthy (%)	1 v. 2		2. Older, optimal healthcare access (%)	2 v. 3		3. Younger, elevated health symptoms (%)	1 v. 3		Main findings
		*χ* ^2^	*V*		*χ* ^2^	*V*		*χ* ^2^	*V*	
Registered as an organ donor	35.3	**83.8**	**0.08**	47.5	**7.4**	**0.03**	43.7	**35.7**	**0.06**	Fewest registered in Profile 1
Have not gotten around to registering	44.4	**81.2**	**0.11**	28.6	0.7	0.01	24.0	**67.5**	**0.10**	Profile 1 is most likely to procrastinate registration
I do not trust organ donation	27.0	**185.2**	**0.17**	9.6	**114.7**	**0.13**	24.0	3.8	0.02	Younger groups (1 and 3) are most distrusting
My religion does not permit it	12.0	**56.9**	**0.09**	4.9	**29.0**	**0.07**	10.1	2.1	0.02	Younger groups (1 and 3) report that religion is an obstacle to registration
I want my body to be whole after I die	26.7	**15.6**	**0.05**	20.8	**4.4**	**0.03**	24.0	3.2	0.02	Younger groups (1 and 3) prefer to preserve the body
I do not think anyone would want my organs	10.5	**155.6**	**0.15**	28.9	**40.5**	**0.08**	18.6	**46.1**	**0.08**	Profile 1 is most likely to underestimate organ demand
I am getting pressured from someone else in my life not to donate	3.7	**10.6**	**0.04**	2.0	**11.2**	**0.04**	4.1	0.1	0.00	Social pressure is rarely reported as an obstacle; younger groups (1 and 3) report it more frequently

*Note:* Bolded cells significantly differ (*p* < 0.05) from the others in the rows based upon *χ*^2^ tests with 1 degree of freedom and Cramer's *V* for effect estimates.

**Table 4:** Results of logistic regression predicting donation registration listed in order by *Z* value effect, and *p* < 0.05 are bolded.

Coefficients	Odds ratio	95% confidence interval	Estimate	Standard error	*Z* value	*p*
Intercept	0.13	0.10–0.17	−2.05	0.13	−15.48	**< 0.01**
Race: White	2.96	2.70–3.27	1.09	0.05	21.72	**< 0.01**
Gender: Male	0.53	0.49–0.58	−0.64	0.04	−14.90	**< 0.01**
Driver's license: Yes	1.55	1.37–1.76	0.44	0.06	6.90	**< 0.01**
Education	1.06	1.04–1.08	0.06	0.01	5.90	**< 0.01**
Health insurance: Yes	1.33	1.19–1.50	0.29	0.06	4.85	**< 0.01**
Income	1.00	1.00–1.00	0.00	0.00	2.25	**0.02**
Hispanic: Yes	0.90	0.80–1.01	−0.11	0.06	−1.85	0.06
Age	0.99	0.98–1.00	−0.00	0.00	−0.63	0.53
Frequency of charitable giving	1.00	0.97–1.04	0.00	0.02	0.08	0.94

## Data Availability

Data from this report can be found at the Center for Open Science: https://osf.io/d4vsm/files/osfstorage.

## Supporting Information

(a) A list of all continuous and categorical variables used as indicators for the latent profile analysis, and distal outcomes.

(b) Fit indices of the stepwise latent-profile modeling. The bolded row indicates the fit indices of the final model; all LMRT and BLRT *p*s < 0.001.
