# Supplementary material for: Latent Profiles of Deceased Organ Donation Registrants and Nonregistrants in the United States
Source: J Transplant. 2025 Jul 18;2025:4446435. doi: 10.1155/joot/4446435 (PMC12297150; doi:10.1155/joot/4446435)
Supplement: Supporting Information — Additional supporting information can be found online in the Supporting Information section. [file 4446435.f1.docx]

**Supplemental Material**. (a) a list of all continuous and categorical variables used as indicators for the latent profile analysis, and distal outcomes.

| Variable type | Survey grouping | Variable name |
| --- | --- | --- |
| *Continuous indicator* | Demographics | Age |
|  |  | Income |
|  |  | Education |
|  | Health and wellbeing | Ease of healthcare access |
|  |  | Healthcare satisfaction |
|  |  | Frequency of medical contact |
|  |  | Chronic pain (PEG-3) |
|  |  | Depression (PHQ-2) |
|  |  | Anxiety (GAD-2) |
|  | Other | Charitable giving |
| *Categorical indicator* |  | State incentive for living donation |
|  |  | Possess driver's license |
|  |  | Medically insured |
| *Distal outcomes* | Demographics | Race |
|  |  | Ethnicity |
|  |  | Gender |
|  | Organ donation | Registration |
|  | Reasons for not donating | “Haven't gotten around to registering” |
|  |  | “I don't trust organ donation” |
|  |  | “My religion doesn't permit it” |
|  |  | “I want my body to be whole after I die” |
|  |  | “I don't think anyone would want my organs” |
|  |  | “I am getting pressured from someone else in my life not to donate” |
| **Supplemental Material**. (b) fit indices of the stepwise latent-profile modelling. | | |

| Profiles | AIC | BIC | Δ BIC | SABIC | Δ SABIC | % of smallest profile | Entropy |
| --- | --- | --- | --- | --- | --- | --- | --- |
| 1 | 429692.8 | 429905 |  | 429813 |  |  |  |
| 2 | 415160.8 | 415512 | -14393.1 | 415359 | -14453.4 | 24.9 | 0.93 |
| **3** | **407468.9** | **407959** | **-7552.94** | **407746** | **-7613.32** | **22.7** | **0.851** |
| 4 | 394539.8 | 395139 | -12819.4 | 394879 | -12867.1 | 21.7 | 0.799 |
| 5 | 391889.1 | 392620 | -2519.02 | 392303 | -2576.22 | 8.5 | 0.82 |

*Note.* **Bolded** row indicates fit indices of the final model; all LMRT and BLRT *p*s < 0.001.

**Conflicts of interest statement**

BP is a member of the scientific and ethics advisory board for Procure OnDemand. The other authors report no financial conflicts of interest.
